# Improving the repair mechanism and miRNA expression profile of tibial defect in rats based on silent information regulator 7 protein analysis of mesenchymal stem cells

**DOI:** 10.1080/21655979.2022.2027066

**Published:** 2022-02-09

**Authors:** Rui Chen, Haizhou Huang, Li Liang, Weibin Zhang, Yingjie Zheng, Dehong Fu, Shibang Lin

**Affiliations:** Orthopaedic Trauma, Maoming People’s Hospital, Maoming City, China

**Keywords:** SIRT7 protein, BMMSCs, tibial defect model, tibial defect, miRNA

## Abstract

The aim of this study was to verify the role of Silent Information Regulator 7 (SIRT7) in improving the repair mechanism of bone marrow mesenchymal stem cells (BMMSCs) and the expression of microribonucleic acid (miRNA). Human BMMSCs were extracted from patients with femoral fractures, and the proliferation activity of human BMMSCs before and after knockout SIRT7 and the expression levels of bone-related genes and proteins were compared. Thirty-two 8-week-old male Sprague-Dawley (SD) rats were randomly divided into a blank group, a chitosan scaffold group, a control group, and a silence information regulator knockout group 7 (n = 8). In addition to the blank group, the chitosan scaffold, the green fluorescent protein (GFP) transfected stem cell composite chitosan scaffold, and the SIRT7 knockout stem cell composite chitosan scaffold were implanted in the other three groups, respectively. The X-rays and small animal in vivo three-dimensional tomography (Micro-CT) were adopted to quantitatively analyze the volume fraction, the number of trabeculae, and the connection density. Compared with the other three groups, the bone defect was formed more in the medullary mesenchymal stem cell knockout group, and the bone volume fraction, number of trabeculae and connection density were significantly increased (*P* < 0.05). MiR-98-5p can significantly promote the formation of bone molecules and bone structure in rats (*P* < 0.05). Human BMMSCs combined with chitosan scaffold can accelerate the repair of tibial defects. MiR-98-5p targeting and regulating bone formation gene (CKIP-1) could significantly improve the process of osteogenesis in rats.

## 1. Introduction

High energy trauma, bone tumor, infection caused by bone defects, and other common orthopedic clinical diseases have always been an important issue in modern medical care. For 5 cm of bone defects, bone transport technique, membrane induction technique, vascularized fibula transplantation and allograft transplantation are generally used in clinics. However, many patients need to be treated by stages, the treatment period is long, the blood supply area is damaged, and the operation is complicated and the complications are easy to occur [1]. Therefore, how to treat bone defects is an urgent problem to be solved. The repair of bone generally needs to go through four stages, namely, the stage of hematoma inflammation, the stage of fibrocartilaginous callus formation, the stage of hard callus formation and the stage of bone remodeling. With the rise and development of bone tissue engineering, this technology has become one of the most potential methods to repair bone defects [2]. At present, the methods of bone tissue engineering mainly include controllable release of growth factors, microsurgical techniques combined with bone tissue engineering, cell co-culture, seed cell and biological scaffold complex. Bone marrow mesenchymal stem cells (BMMSCs) are the most widely used seed cells in bone tissue engineering field. They have a wide range of sources, are easy to obtain, have a high degree of self-renewal, and offer extremely strong multi-directional differentiation potential characteristics. They can not only differentiate into connective tissue, such as bone, cartilage, and fat [3], but also induce differentiation into endothelial cells to form vascular-like tissues [4]. Many growth factors and cytokines can promote BMMSCs differentiation, and enhance migration and homing of stem cells to promote bone repair [5–8].

BMMSCs not only promote bone repair through differentiation but also through a series of immunomodulatory effects [9,10]. The Sirtuin protein family is a nicotinamide adenine dinucleotide (NAD+) dependent protein deacetylase and/or adenosine diphosphate (ADP) ribosyltransferase. It plays an important role in different cellular processes, such as chromatin silencing, cell cycle regulation, cell differentiation, cell stress response, and aging [11; 12, 13]. The Sirtuin is a highly conserved deacetylase from bacteria to humans. There are seven recognized members of the human Sirtuin protein family: Silent information regulator 1–7. The knockout Silent Information Regulator 3 (SIRT3) can reduce mitochondrial function and osteogenic differentiation [14,15], and Silent Information Regulator 1 (SIRT1) agonist resveratrol can also promote osteogenesis of human BMMSCs by activating Runt-related transcription factor 2 (RUNX2) [16–21]. The knockout Silent Information Regulator 7 (SIRT7) can enhance hypoxia-inducible factor 1 (HIF-1) and hypoxia-inducible factor 7 (HIF-7) transcriptional activity and increase intracellular HIF-1α and HIF-2α protein levels by inhibiting ubiquitination [22]. HIF-1α regulation of transcription promotes the expression of vascular endothelial growth factor A (VEGF- A) [23,24]. SIRT7 is suspected to play an important role in the osteogenic differentiation of BMMSCs. Meanwhile, in recent years, studies have confirmed that casein kinase 2 interacting protein-1 (CKIP-1) is involved in many important cellular pathway processes, including most physiological processes of cells. In most studies, CKIP-1 is regarded as an important regulator of cell osteogenic differentiation [25,26]. For example, reducing CKIP-1 expression by silencing technology can significantly increase the overall bone mineral density and bone structure of C57BL/6 mice [27]. Micro-Ribonucleic Acid (miRNA), as an early discovered small-molecule mRNA regulator, has been proved to play a key regulatory role in the process of cell osteogenic differentiation. It has been reported that miR-let-7i-5p is involved in a variety of cell regulatory processes, such as adipocyte differentiation and cancer cell proliferation [28]. Some studies have found the downstream target gene silencing information regulator 3 (Sirtuin3, SIRT3) of miR-98-5p to improve cell damage caused by oxidative stress [29]. In addition to the above methods, some scholars have used radiological and histological methods to study the effect of tranexamic acid in the healing of closed femoral fractures in experimental rat models. The results show that tranexamic acid can effectively accelerate early bone formation and fractures healing [30].

The effect of SIRT7 knockout on human BMMSCs is determined by comparative analysis of the value-added activities of BMMSCs and the expression levels of bone-related genes and proteins. Using the rat tibial defect model, the parameters such as volume fraction, number of trabeculae and connection density are analyzed quantitatively by X-ray and Micro-CT platform to study the effect of human BMMSCs combined with chitosan scaffold on improving the repair mechanism of tibial defect in rats. In addition, bioinformatics analysis, quantitative reverse transcription polymerase chain reaction (qRT-PCR) and other methods are used to preliminarily screen miRNA related to the osteogenesis process in rats, which provide a bioinformatics basis for subsequent validation. In view of the basics of theoretical research, this study assumed that SIRT7 had an active role in tibial defect repair and had the ability to regulate certain tiny ribonucleotides to accelerate the repair. The objective of this research was to explore the role of BMMSCs in improving the repair mechanism of tibial defects and the expression of microribonucleic acid; the goal of this research was to extract human BMMSCs from patients with femoral fractures and analyze the silent information using the rat tibial defect model SIRT7 before and after the proliferation activity of BMMSCs and the expression levels of bone-related genes and proteins.

## 2. Materials and method

### 2.1. Construction of rat tibial defect model

Thirty-two 8-week-old male Sprague-Dawley (SD) rats (with a weight of around 200 g) were purchased from Tianjin and were numbered and randomly divided into four groups: a blank group, a chitosan scaffold group, a control group, and a knockout SIRT7 group (n = 8). The SD rats were anesthetized with 0.3% pentobarbital sodium (30 mg/Kg) [31]. After anesthesia, the skin around the left knee joint was cut open, and the intramedullary needle (1.2 mm diameter stainless steel syringe needle) was inserted into the tibial medullary cavity through the lateral platform of the tibial plateau [32]. A unilateral tibial defect with a diameter of 1.5 mm was drilled with a dental drill 5 mm beyond the growth plate. The rats in blank group were not implanted with anything, the defects of chitosan scaffold group were only implanted with chitosan scaffold, the defects of control group were implanted with stem cell compound chitosan scaffold transfected GFP (Shanghai Gemma pharmaceutical technology, Co. Ltd.), and the defect of experimental group was implanted with stem cell complex chitosan scaffold of knockout SIRT7. After operation, the subcutaneous fascia and skin were sutured after cleaning, and the left tibial specimen was obtained by euthanasia after 6 weeks of standard feeding conditions. The parameters such as volume fraction, number of bone trabeculae and connection density were quantitatively analyzed by X-ray and Micro-CT platforms (All animal treatment and experimental handling were in accordance with the national laboratory animal code, and the experimental implementation process had been approved by the Ethics Committee).

### 2.2. Extraction and SIRT7 knockout identification of human BMMSCs

Human BMMSCs were extracted from the bone marrow of two patients with femoral fracture (all male, 22 and 23 years old), and the culture and proliferation of the extracted human BMMSCs were detected. Among them, human BMMSCs were resuspended in Dulbecco’s modified eagle medium (DMEM)/F12 containing fetal bovine serum (FBS). They were counted and placed in a 75 mL culture flask with 1*10^6^ /mL, and then incubated in DMEM/F12, supplemented with 10% FBS, 100 U/mL penicillin/and 100 μg/mL streptomycin was cultured in an incubator at 5% CO_2_ and 37°C saturated humidity. The culture medium was changed at 72 hours, both cells were digested with trypsin and sub-cultured after 7 days (Shanghai Jinma Pharmaceutical Technology Co., Ltd.). Then, the third-generation human BMMSCs were transfected with lentivirus to knock out SIRT7 gene. When the third generation of human BMMSCs grew and fused to about 50%, lentivirus particles and transfection agents (polybrene, 2.5 μg/mL) were added to the complete medium, and the plural number of infections was 50 [33]. After 12 hours, the culture medium was changed, and more than 95% of the cells still survived. After the cells grew and fused to about 90%, the cells were sub-cultured. Puromycin (1 μg/mL) was added to the sub-cultured cell culture medium, and the culture medium was changed in 2 days. After the cells grew and fused to about 90%, the cells were sub-cultured and used for subsequent research.

The Cell Counting Kit-8 (CCK-8) method (purchased in Dojindo company, Japan) was used to detect the value-added activity of human BMMSCs before and after knockout SIRT7, as well as the expression level of osteogenic related genes and proteins after knockout to meet the minimum standard of human pluripotent mesenchymal stem cells proposed by the international association of cell therapy. The human BMMSCs transfected with lentivirus were mixed with 50,000 cells per scaffold and added to the chitosan scaffold in a complete medium of 50 μL (Seye, Guangzhou, China). The culture was continued for subsequent experiments (all experiments involving the acquisition of human tissue specimens had been approved by the ethics committee, each tissue specimen source had been approved with the consent of the patient and their family, and an informed consent had been signed).

### 2.3. Detection of alkaline phosphatase (ALP) and calcium nodules in human BMMSCs

ALP activity and the formation of calcium nodules are the signs of early osteogenic differentiation. During the detection of ALP level, human BMMSCs were inoculated into 12-well plates, and osteogenic differentiation was induced to days 4 and 8. After the culture medium was discarded, the human BMMSCs were washed with phosphate buffer saline (PBS) 3 times, and fix with 4% paraformaldehyde for 15 min. After the PBS was cleaned 3 times and 500 μL pre-prepared ALP dye was added, the BMMSCs were incubated in the dark at room temperature for 30 min to discard the ALP dye [34]. After PBS cleaning for 3 times, it should record the value at the detection wavelength of 520 nm in the spectrophotometer.

During the detection of calcium nodules, human BMMSCs were inoculated on 12-well plates by alizarin red staining, and osteogenic differentiation was induced until the 4th and 8th days. After the culture medium was discarded, the BMMSCs were rinsed with PBS 3 times, and fixed with 4% paraformaldehyde for 15 min. They were washed with PBS 3 times, and alizarin red was added and dyed for 30 min. After they were washed 3 times, the BMMSCs were observed under an inverted optical microscope. Ten percent cetane chloride was added to each well for decolorization for 1 h. 200 μL decolorizing solution in each well was added to a 96-well plate. Finally, the value of calcium nodules at 560 nm was detected by spectrophotometer.

### 2.4. Screening of miRNA that regulate bone formation genes (CKIP-1)

The study expects to further understand the mechanism of osteogenic differentiation and analyzes the expression changes of osteogenic-related miRNA from the perspective of bioinformatics. By searching several standard miRNA online databases (Target Scan: www.targetscan.org/vert_72, Pic Tar: https://pictar.mdc-berlin.de/; mi RBase: www.mirbase.org), the target miRNA selection of CKIP-1 was predicted to have high thermal stability and high conservation miRNA in human, mouse, rat and other species [35]. Three rno-miRNA with complementary binding sites to CKIP-1 3ʹUTR were preliminarily screened: rno-let-7d-5p, rno-mir-98-5p, and rno-mi R-129-5p (Table 1). Human BMMSCs were induced to differentiate. The expression changes of three candidate miRNAs before and after osteogenic induction were detected by RT-PCR. Primary screening improved the miRNA associated with osteogenesis in rats.

**Table 1.** miRNA sequence table

Name	CKIP-1 3ʹUTR	(5’-3’)
rno-let-7d-5p	76–82 /2882-2888	AGAGGUAGUAGGUUGCAUAGUU
rno-mi R-129-5p	86–92	CUUUUUGCGGUCUGGGCUUGC
rno-mi R-98-5p	76–82/2882-2888	UGAGGUAGUAAGUUGUAUUGUU

### 2.5. Western blot analysis

The expression levels of SIRT7 protein in the 3rd and 9th generation BMMSCs of different groups were detected. After the cells were washed three times with the precooled PBS, a small amount of cell lysate was added to cover the cell surface, lysed for 5 minutes, centrifuged to take the supernatant, and transferred to another 1.5 mL EP tube. The above operations were carried out at 4°C. 4 µL supernatant was put into 1 mL of Coomassie brilliant blue (PIERCE company, USA) prepared before. After shaking, 200 µL/well was taken and placed in 96-well plate. Each sample was provided with 3 multiple wells. The absorbance of each well was measured with an enzyme labeling instrument at 570 nm wavelength. A standard curve (y = ax+b) was drawn, based on which the concentration of sample protein to be tested was calculated. Subsequently, it should perform the sodium dodecyl sulfate polyacrylamide gel electrophoresis (SDS-PAGE). After SDS-PAGE, the concentrated gel was cut off, and a polyvinylidene fluoride (PVDF) membrane with appropriate size was prepared. The wet transfer method was adopted, and the film was turned at 80 V constant pressure for 120 minutes [36]. The membrane was taken out, immersed in 5% skimmed milk, and incubated at room temperature for 1 hour. The antibody was diluted and incubated according to the proportion in the manual, shaken at 4°C and overnight. 1 × Tris-Buffered Saline Tween-20 (TBST) film was washed for 10 min × 4 times. The corresponding horseradish peroxidase-labeled secondary antibody (Beijing Zhongshan Jinqiao Biotechnology Co., Ltd., China) (1:2000 dilution) was added and incubated at room temperature for 1 h. The 1 × TBST film was reused to wash 10 min × 4 times. The liquid A and liquid B (1:1) of the developing solution were mixed, evenly added to the surface of PVDF film, and exposed and stored twith digital multifunctional image-enhanced chemiluminescence system (Shanghai Tianneng Technology Co., Ltd., China) to observe the results. The housekeeping gene glyceraldehyde 3-phosphate dehydrogenase (GAPDH) was used as an internal reference for calibration.

### 2.6. Histological staining

After 6 weeks, the left tibia specimen was obtained by euthanasia, fixed with 4% paraformaldehyde for 2 days, decalcified with 10% ethylenediaminetetraacetic acid disodium solution, and replaced with ethylenediaminetetraacetic acid disodium solution once a week for 8 consecutive weeks. After embedded in paraffin, the sectioning process (3 mm) was performed. After sectioning, the sections were stained with eosin-hematoxylin, and the slides were dehydrated with alcohol for mounting, and were observed and recorded under a microscope [37].

### 2.7. Statistical analysis

SPSS17.0 software was used for statistical analysis in experiment. All experiments were performed at least three times, and the data results were expressed mean ± standard deviation. Statistical significance: t-test was used to compare pairwise data, univariate analysis of variance was used for comparison among three groups and above data sets, and Bonferroni test was used for pairwise comparison. *P* < 0.05 was considered to be a statistically significant difference, and *P* < 0.01 was a very statistically significant difference.

## 3. Results

The objective of this research was to explore the role of SIRT7 in improving the repair mechanism of BMMSCs in tibia defect and the expression of microribonucleic acid. The human BMMSCs were extracted from patients with femoral fractures, and the effects of SIRT7 on the proliferation activity of human BMMSCs and the expression levels of bone-related genes and proteins were analyzed. In addition, the rat tibia defect model was prepared, and the rats were randomly divided into a blank group, a chitosan stent group, a control group, and a SIRT7 group (n = 8). The X-ray and small animal in vivo three-dimensional tomography system were adopted for quantitative analysis of volume fraction, number of trabeculae, and connection density.

### 3.1. The endogenous expression of SIRT7 in human BMMSCs and the effect verification of knockout SIRT7

**Figure 1.**
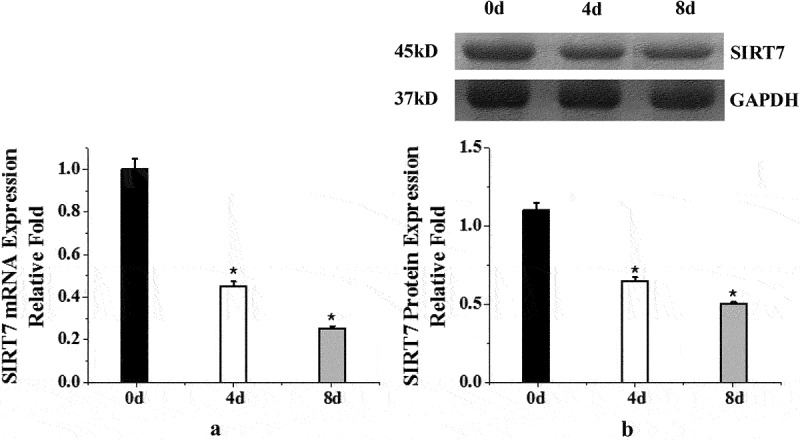
Endogenous expression of human BMMSCs (a: mRNA expression changes of human BMMSCs in osteogenic differentiation; b: quantitative analysis of protein expression. (*: compared with 0d, *P* < 0.05)).

In order to observe the endogenous expression of SIRT7 during osteogenic differentiation of human BMMSCs, the expression levels of information RNA and protein were detected before osteogenic differentiation (0d) and 4d and 8d after osteogenic differentiation. Figure 1 shows the endogenous expression of SIRT7 in human BMMSCs during osteogenic differentiation. The results showed that the expression levels of SIRT7 mRNA and protein in human BMMSCs on the 4th and 8^th^ days after osteogenic differentiation were significantly lower than those before osteogenic differentiation (0d) (*P* < 0.05).

**Figure 2.**
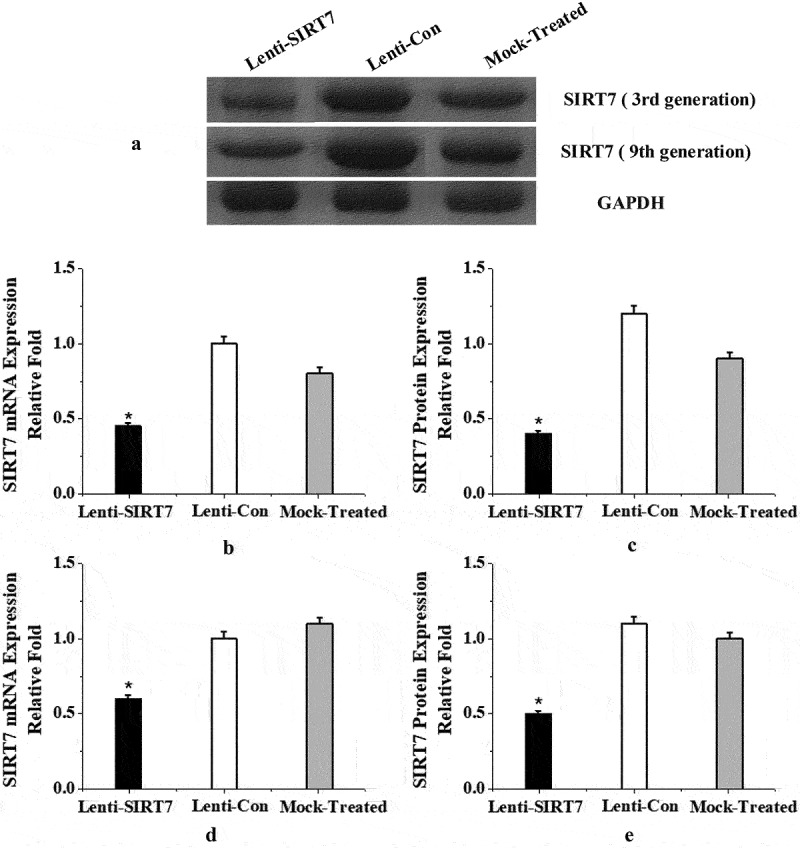
SIRT7 knockout effect of BMMSCs in the 3rd and 9th generation (a: Western blot electrophoresis; b, c: mRNA and protein levels of SIRT7 in the 3rd generation after transfection; d, e: mRNA and protein levels of SIRT7 in the 9th generation after transfection. Lenti-SIRT7: SIRT7 knockout group; Lenti-Con: control group; Mock-Treated: simulation group (not added with any lentivirus but added with a lentivirus transfection agent). (*: compared with control group, *P* < 0.05)).

In the experiment, BMMSCs of the 3rd generation were transfected with lentivirus to knockout the SIRT7 gene, and the expression of SIRT7 in lentivirus-transfected cells was detected by PCR and protein electrophoresis. The mRNA and protein expression of stem cell SIRT7 in the SIRT7 knockout group were significantly lower than those in the control and simulation groups (*P* < 0.05). In order to verify the knockout efficiency during the passage of lentiviruses after transfection, the study tested the mRNA and protein expression levels of the 9th generation SIRT7. The results showed that the knockout SIRT7 group can effectively knock down the silence information regulator 7 in human BMMSCs, and it was significantly lower than the control group and the simulation group (*P* < 0.05) (Figure 2).

### 3.2. Value-added activity of human BMMSCs and expression level of osteogenic related genes and proteins after knockout SIRT7

**Figure 3.**
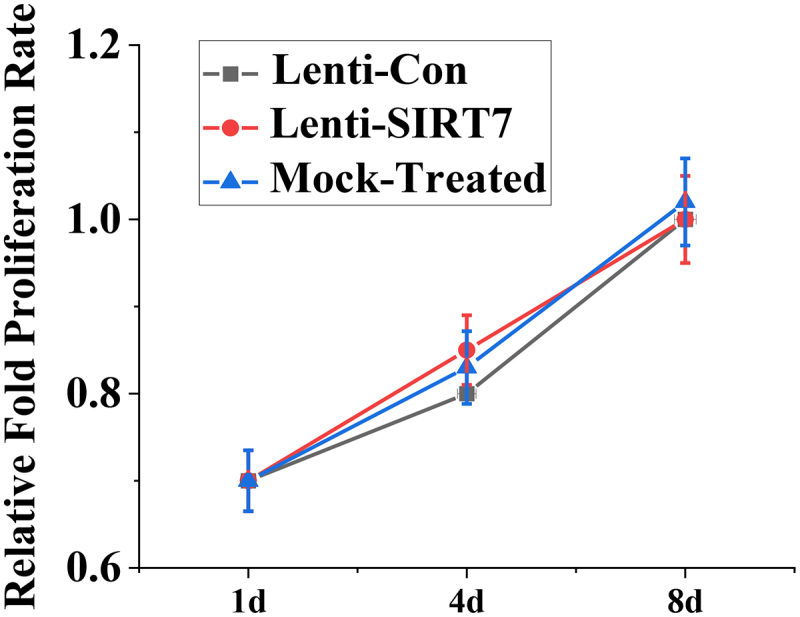
The value-added activity detection of human BMMSCs (Lenti-SIRT7: SIRT7 knockout group; Lenti-Con: control group. (*: compared with control group, *P* < 0.05)).

The value-added activity of human BMMSCs before and after knockout SIRT7 was detected by CCK-8 method, and Figure 3 shows the value-added activity results of human BMMSCs. On the 1st, 4th, and 8th days after human BMMSCs transfection with lentivirus, there was no significant difference in the proliferative activity between the SIRT7 knockout group and the control group. It is suggested that the knockout SIRT7 gene in human BMMSCs does not affect the proliferation ability of stem cells.

**Figure 4.**
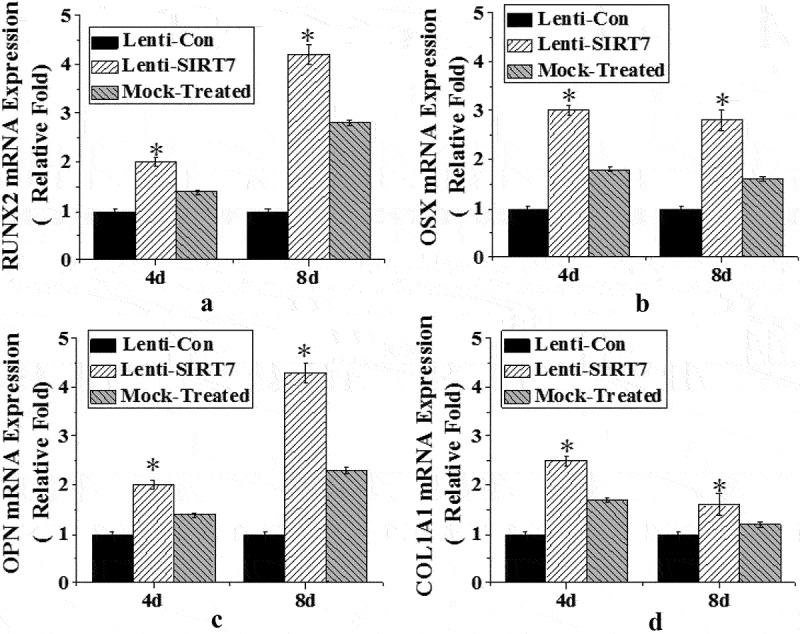
The mRNA expression levels of various genes in osteoblasts (Lenti-SIRT7: SIRT7 knockout group; Lenti-con: control group; Mock-Treated: simulation Group (not added with any lentivirus but added with a lentivirus transfer agent). a. RUNX2 gene; b. OSX gene; c. OPN gene; d. COL1A1 gene (*: compared with the control group, *P* < 0.05)).

To investigate the effect of SIRT7 knockout on osteogenic differentiation, the expression levels of osteoblast-related genes and proteins RUNX2, osterix (OSX), osteopontin (OPN), and type 1 collagen (COL1A1) were detected. The results showed that the mRNA levels of RUNX2, OSX, OPN, and COL1A1 in the stem cells in the SIRT7 knockout group were significantly higher than those in the control group at the 4th and 8th day after osteogenic differentiation. However, there was no significant difference in the mRNA levels of RUNX2, OSX, OPN, and COL1A1 between the simulation group and the control group (Figure 4).

### 3.3. Changes of ALP activity and calcium nodule level after SIRT7 knockout

**Figure 5.**
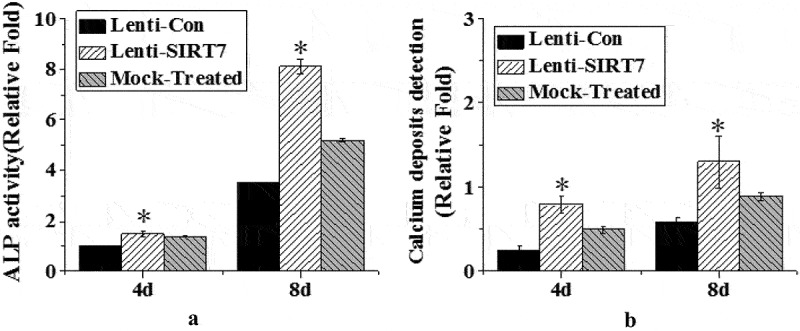
Changes of ALP activity and calcium nodule level in osteoblasts (Lenti-SIRT7: SIRT7 knockout group; Lenti-con: control group; Mock-Treated: simulation Group (not added with any lentivirus but added with a lentivirus transfer agent); a. ALP activity; b. calcium nodule level (*: compared with the control group, *P* < 0.05)).

ALP activity and calcium nodule levels were detected on the 4th and 8th day of osteogenic differentiation after silencing information regulator 7 knockout (Figure 5). On the 4th and 8th days of osteogenic differentiation after SIRT7 knockout, the stem cells showed stronger enzyme activity, which was statistically different from the control group (*P* < 0.05), but there was no significant change in the simulation group compared with the control group (Figure 5(a)). Meanwhile, the level of stained calcium nodules was detected. The stem cells knockout of SIRT7 produced more calcium nodules than the stem cells in the control group (Figure 5(b)). The above results showed that knocking out SIRT7 gene in human BMMSCs could promote the process of osteogenic differentiation.

### 3.4. The accelerated repair of tibial defects in rats by the combination of knockout SIRT7 human BMMSCs and chitosan scaffolds

**Figure 6.**
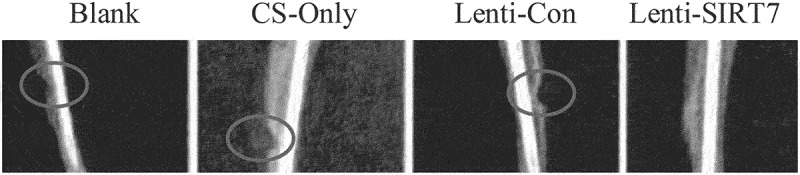
X-ray during 6 weeks after tibial defect in rats (Blank: blank group; CS-Only: chitosan scaffold group; Lenti-con: control group; Lenti-SIRT7: knockout SIRT7 group. The circle showed the degree of bone defect.).

**Figure 7.**
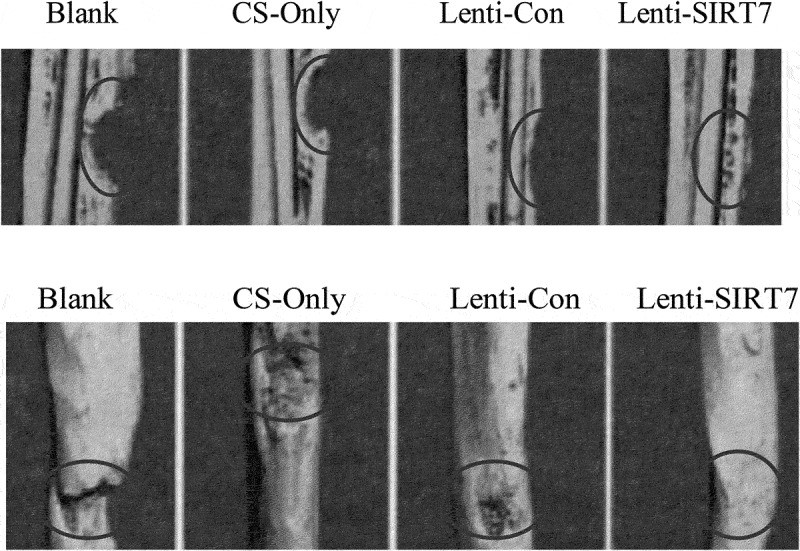
Missing surface and reconstructed image of Micro-CTD (Blank: blank group; CS-Only: chitosan scaffold group; Lenti-con: control group; Lenti-SIRT7: knockout SIRT7 group. The circle shows the degree of bone defect.).

**Figure 8.**
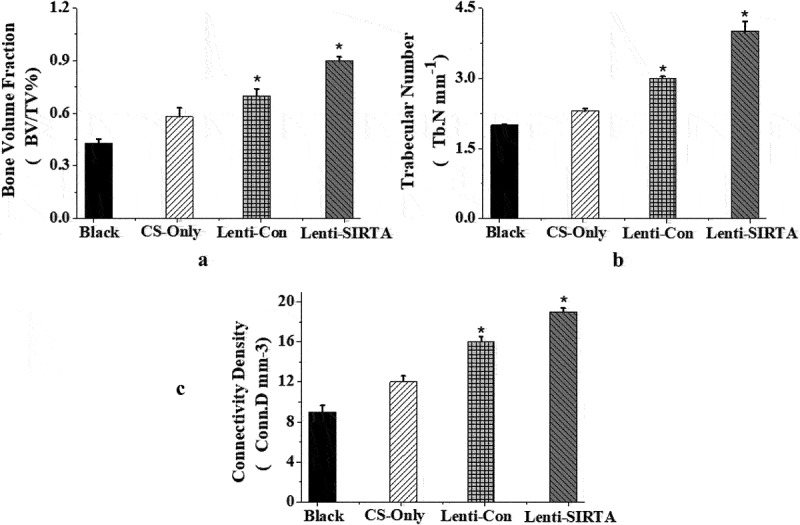
Quantitative analysis of Micro-CT (Blank: blank group; CS-Only: chitosan scaffold group; Lenti-con: control group; Lenti-SIRT7: knockout SIRT7 group. a. Bone volume fraction; b. trabecular number; c. connectivity density; (*: compared with Black, *P* < 0.05)).

The X-ray results showed that the cortical bone defects were still clear in the blank group at the 6th week after surgery, and the tibial cortical bone defects were still visible in the stent-only group and the control group, but smaller than those in the blank group. The cortical bone defects of SIRT7 group were filled with callus, and the cortical bone defects almost disappeared (Figure 6). Micro-CT results also showed that more new bones were formed at bone defects in the knockout SIRT7 group, while significant bone defects were found in the other three groups, with the blank group being the most obvious (Figure 7). The quantitative analysis of Micro-CT indicated that the bone volume fraction, bone trabecular number, and connection density in the knockout SIRT7 group were significantly higher than those in the blank group, and were the highest among the four groups (Figure 8).

### 3.5. CKIP-1 genes formation of miR-98-5p regulation of bone

**Figure 9.**
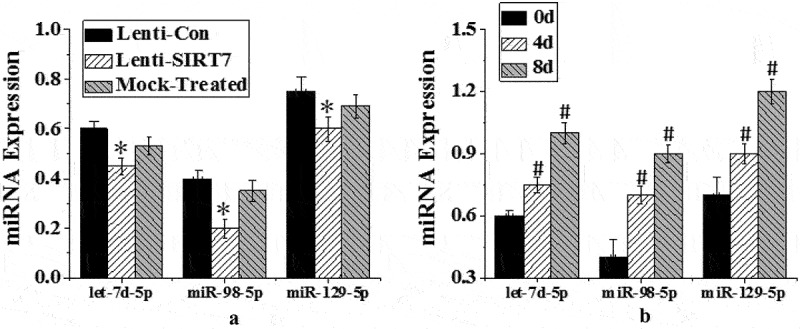
Expression changes of different miRNAs in osteogenic differentiation (a. different groups; b. different induced differentiation time; (*: *P* < 0.05 compared with the control group; #: *P* < 0.05 compared with that before osteogenic differentiation (0d)).

Three rno-miRNA with complementary binding sites to CKIP-1 3ʹUTR were preliminarily screened: rno-let-7d-5p, rno-mir-98-5p, and rno-mi R-129-5p. RT-PCR was used to detect the expression changes of three candidate miRNAs before osteogenic differentiation (0d) and the 4 and 8 days after osteogenic differentiation. The expression of let-7d-5p, miR-129-5p, and miR-98-5p increased significantly on the 4th and 8th days after osteogenic differentiation (*P* < 0.05), while the level of miR-98-5p changed more significantly. The expression of three candidate miRNAs in the control group, SIRT7 knockout group, and simulation group was detected. The expression levels of let-7d-5p, miR-129-5p, and miR-98-5p in human BMMSCs in SIRT7 knockout group decreased significantly compared with the control group (*P* < 0.05), while the expression levels of three candidate miRNAs in the simulation group did not decrease significantly (Figure 9).

### 3.6. Staining results of tissue sections

**Figure 10.**
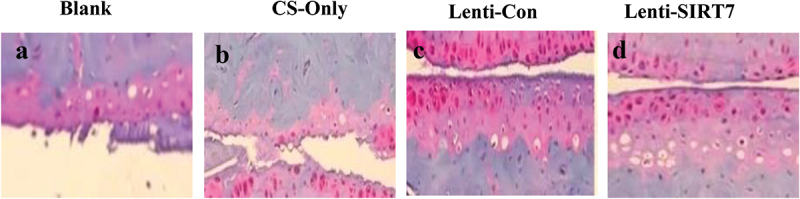
Eosin-hematoxylin staining results of tissue sections of tibial defects 6 weeks postoperatively (a. blank control group; b. chitosan scaffold group; c. control group; d. knockout SIRT7 group).

The staining results of tibial defect tissue slices in the 6th week after surgery were compared (Figure 10). It can be observed that there was only a small amount of new bone tissue in the blank group; obvious new bone tissue was visible in the chitosan scaffold group and control group; and the knockout SIRT7 group. In addition, the bone defect was repaired obviously, and there was a lot of new bone tissue.

## 4. Discussion

Some scholars found that SIRT7 gene knockout significantly enhanced osteoblast-specific gene expression, ALP activity, and mineral deposition in vitro. SIRT7 plays an important role in the osteogenic differentiation of BMMSCs, partly by activating Wnt/β- Catenin signaling pathway. The combination of SIRT7 knockout human BMMSCs with chitosan scaffolds can significantly promote bone formation in the rat tibial defect model [38,36]. Human BMMSCs used in experiment could be adherent culture, and accorded with the distribution of surface markers; at the same time, it could be differentiated by three lines, and accorded with the minimum standard of appraising pluripotent mesenchymal stem cells published by the international association of cell therapy in 2006, which laid a good foundation for subsequent experimental research. By knocking out SIRT7 gene, its role in osteogenic differentiation of human BMMSCs had been studied. Endogenous SIRT7 expression was found to decrease gradually during osteogenic differentiation; hence, lentivirus transfection technique was used to knockout SIRT7 genes in mesenchymal stem cells to accelerate the process of osteogenic differentiation. Moreover, knockout SIRT7 did not affect the proliferation ability of BMMSCs, and these results suggested that knockout SIRT7 could promote osteogenic differentiation of human BMMSCs in vitro. It had been reported that porous chitosan-alginate scaffold had bone conductivity and could improve the healing of skull defects [39], and chitosan fiber could promote the proliferation, osteogenic differentiation and bone regeneration of rabbit BMMSCs [40]. Loose chitosan scaffolds were also used to combine human BMMSCs to promote the repair of tibial bone defects in rats in this experiment. It was found that the repair effect of bone defects was best in the group of SIRT7-knocked stem cells combined with chitosan scaffold group. Meanwhile, the expression levels of let-7d-5p, miR-129-5p, and miR-98-5p in human BMMSCs in SIRT7 knockout group decreased significantly, and the level of miR-98-5p changed more significantly. It is necessary to further verify and analyze the targeted regulation of SIRT7 gene by miR-98-5p to significantly improve the osteogenesis process of rats. In addition to the SIRT7 explored in this article, some scholars have used bone morphogenetic protein to induce new bone formation, and combined it with a variety of carriers (natural and synthetic polymers, inorganic materials, and their combinations), in mice, tested in various models of non-human primates, such as rats, rabbits, and dogs [41]. Compared with the method proposed in this study, this method has slightly different bone defect repair results, but the advantages and disadvantages of the two methods need to be improved experimentally.

The preliminary study revealed the effect of SIRT7 on the osteogenic differentiation of mesenchymal stem cells and laid a foundation for further research, but there were still some limitations in this experiment. Overexpression SIRT7 in human BMMSCs also needed to be investigated for its effect on osteogenic differentiation and further explore the related signaling pathways involved in SIRT7 regulation BMMSCs osteogenic differentiation.

## 5. Conclusion

The aim of the experiment is to verify the role of SIRT7 protein in BMMSCs in improving the repair mechanism and miRNA expression of tibial defect in rats. Based on the above analysis, the main results obtained were as follows:

The mRNA and protein expression of human BMMSCs decrease significantly with the increase in osteogenic differentiation time, and BMMSCs transfected with lentivirus in the 3^rd^ generation can knock out SIRT7 gene better. In addition, the knockout efficiency of SIRT7 gene in human BMMSCs was still maintained in the process of passage, with the increase in osteogenic differentiation time. The knockout of SIRT7 genes in human BMMSCs did not affect the proliferation ability of stem cells. The mRNA levels of RUNX2, OSX, OPN, and COL1A1 in stem cells of knockout stem cells increase significantly. Quantitative analysis of X-ray, Micro-CT, and Micro-CT showed that there had been more bone formation in bone defects in the knockout SIRT7 human BMMSCs, bone volume fraction, bone trabecular number, and junction density were also significantly increased. That is, the human BMMSCs combined with chitosan scaffolds after SIRT7 knockout accelerated the repair of tibial defects in rats. MiR-98-5p targeting CKIP-1 genes could significantly improve the osteogenesis process in rats.
